# Extreme climatic events down-regulate the grassland biomass response to elevated carbon dioxide

**DOI:** 10.1038/s41598-018-36157-x

**Published:** 2018-12-10

**Authors:** Naiming Yuan, Gerald Moser, Christoph Mueller, Wolfgang A. Obermeier, Joerg Bendix, Jürg Luterbacher

**Affiliations:** 1 Department of Geography, Climatology, Climate Dynamics and Climate Change, Justus-Liebig University Giessen, Senckenbergstr. 1, 35390 Giessen, Germany; 2 CAS Key laboratory of Regional Climate-Environment for Temperate East Asia, Institute of Atmospheric Physics, Chinese Academy of Sciences, Beijing, 100029 China; 3 Department of Plant Ecology, Justus-Liebig University Giessen, Heinrich-Buff-Ring 26, 35392 Giessen, Germany; 4 School of Biology and Environmental Sciences, University College Dublin, Dublin, Ireland; 5 Faculty of Geography, Laboratory for Climatology and Remote Sensing, Philipps-University of Marburg, Deutschhausstr. 10, Marburg, Germany; 6 Centre for International Development and Environmental Research, Justus-Liebig University Giessen, 35390 Giessen, Germany

## Abstract

Terrestrial ecosystems are considered as carbon sinks that may mitigate the impacts of increased atmospheric CO_2_ concentration ([CO_2_]). However, it is not clear what their carbon sink capacity will be under extreme climatic conditions. In this study, we used long-term (1998–2013) data from a C3 grassland Free Air CO_2_ Enrichment (FACE) experiment in Germany to study the combined effects of elevated [CO_2_] and extreme climatic events (ECEs) on aboveground biomass production. CO_2_ fertilization effect (CFE), which represents the promoted plant photosynthesis and water use efficiency under higher [CO_2_], was quantiffied by calculating the relative differences in biomass between the plots with [CO_2_] enrichment and the plots with ambient [CO_2_]. Down-regulated CFEs were found when ECEs occurred during the growing season, and the CFE decreases were statistically significant with *p* well below 0.05 (t-test). Of all the observed ECEs, the strongest CFE decreases were associated with intensive and prolonged heat waves. These findings suggest that more frequent ECEs in the future are likely to restrict the mitigatory effects of C3 grassland ecosystems, leading to an accelerated warming trend. To reduce the uncertainties of future projections, the atmosphere-vegetation interactions, especially the ECEs effects, are emphasized and need to be better accounted.

## Introduction

Atmospheric carbon dioxide concentration [CO_2_] has increased substantially since industrialization and is projected to rise by 40% from approx. 400 ppm in early 2017 to 550 ppm by 2050 (RCP8.5 scenario)^1,2^. The rising atmospheric CO_2_ concentration contributes largely to global warming, and also stimulates ecosystem productivity^3–5^. It has been estimated that terrestrial ecosystems have sequestered about 25% of the anthropogenic carbon emissions over the past half-century^6^. Therefore, most ecosystems potentially act as carbon (C) sinks and mitigate the effects of increased [CO_2_]^7,8^. Concerns regarding the future capacity of ecosystems as C sinks have been raised due to the negative effects of extreme climatic events (ECEs)^9^. Extreme events such as prolonged heat waves, droughts, and frosts can significantly reduce ecosystem carbon uptake and productivity, thereby influencing the regional carbon cycle^10–13^ and gradually shifting the ecosystems from a C sink towards a C source^10^. Since ECEs have been projected to increase in both frequency and intensity^14,15^, studying the combined effects of ECEs and elevated atmospheric [CO_2_] ([eCO_2_]) on the carbon cycle of ecosystems is important for both understanding mechanisms^16^ and reducing predictive uncertainty^17^.

Elevated [CO_2_] stimulates ecosystem productivity (termed the CO_2_ fertilization effect, CFE) directly through (i) enhanced photosynthesis^3,18^, or indirectly through (ii) reduced stomatal conductance^19–22^ and (iii) reduced respiration^23^. Accordingly, one may expect stronger CFEs at higher temperatures or in drier conditions^24,25^, and the negative effects of ECEs may be ameliorated through improving water use efficiency (WUE)^26^, increasing the plant carbon uptake^27^, and enhancing recovery after ECEs^28^. However, different studies have demonstrated that these theories are not applicable to all ecosystems^29,30^. In some experiments, [eCO_2_] was found to have no alleviating effect against ECEs^31,32^. On the contrary, [eCO_2_] may increase the risk of exposure to ECEs by extending the growing season length^33^. Meanwhile, ECEs can prevent plants from benefiting from [eCO_2_]^34^. The CFEs may be strongest under intermediate environmental conditions and vanish under more extreme weather conditions^35^. The inconsistencies in these results indicate an important role for ECEs in altering CFEs, and emphasize the necessity for more detailed studies, which have thus far been prevented due to the lack of suitable long-term continuous and high quality data^36^.

In this study, we analyzed data from one of the longest Free Air Carbon dioxide Enrichment (FACE) experiments (Gi-FACE, 1998–2013) in the world and studied the combined effects of [eCO_2_] and ECEs on aboveground biomass production. The Gi-FACE experiment was carried out on a permanent grassland in the German federal state of Hesse, near Giessen (50°32′N, 8°41′E) at 172 m a.s.l. (Fig. S1). Three circular plots were subjected to [eCO_2_], while another three circular plots served as controls at ambient [CO_2_] ([aCO_2_]). They were arranged in a randomized block design (three blocks). The CO_2_ fumigation began in May 1998 with an enrichment level of +20% [CO_2_] above the ambient level during daylight hours (Fig. S2). The vegetation comprised species-rich grassland where aboveground grass biomass contributed more than 2/3 of the harvest in most years (Tables S1, S2). Biomass was harvested twice a year before the end of spring (H1) and summer (H2)^37^ (Table S3).

In contrast to recent work by Obermeier *et al*.^35^, that used the summer growing season data from the Gi-FACE only, we here focused on both spring and summer growing seasons. That is, not only the extreme climatic conditions in summer such as drought, heatwaves, etc., were studied, but also the extreme events from spring including frost events were considered. Since changes of magnitude or frequency of extreme events are likely to impair plant production, we here focused on single extreme events rather than estimating the impacts from changes of mean climate conditions. Accordingly, our work gives more direct evidences on how ECEs alter CFEs, which beyond the work by Obermeier *et al*.^35^. The ECEs were determined using various environmental datasets including 2m-air temperature records, precipitation records, soil moisture, etc. We determined extreme dry events for both growing seasons, anomalous cold events including hard frost in spring, and extreme hot events including heat waves in summer. The definitions are provided in the “Material and Method” section, and corresponding figures can be found in the Supplementary Materials (Figs S3–S8). The CFE in this study is represented by the effect size (ES) of the aboveground biomass, which is defined as the relative differences in biomass between the eCO_2_ plots and the aCO_2_ plots. We assumed that the ES of adjacent years under similar growing conditions did not differ significantly, i.e., sudden changes in growing conditions, such as those caused by ECEs, may lead to significant changes in ES. Therefore, by investigating the changes in ES of the biomass in comparison with the previous year we were able to examine the impact of ECEs on the yield stimulating effect of e[CO_2_].

Before the connections between extreme climatic events and the CO_2_ fertilization effects on the aboveground biomass can be studied, we first need to check whether the calculated effect size represented the true CO_2_ effect. By setting a repeated measures analysis of variance (rmANOVA) model with factors time, CO_2_, block, time × CO_2_, and time × block included, the treatment effects for different plant functional groups were studied. As shown in Table 1, there were significant CO_2_ fertilization effects on grass (H1, 1998–2005, 1998–2013; H2, 1998–2005), but the differences of the forbs (incl. legumes) biomass cannot be explained by the [CO_2_] treatment. As a result, when considering total biomass, the treatment effect was only statistically significant for H2 when the second time section (2007–2013) was considered. This is reasonable as large initial biases of the forbs (incl. legumes) existed in the first few years of the experiment. As discussed in^38^, since 1997 before the start of the FACE up to the years before 2006/2007, there were more forbs and legumes harvested in the aCO_2_ plots than in the eCO_2_ plots. Benefited from the higher [CO_2_] in eCO_2_ plots, an increasing trend of ES was observed from 2001 to 2008 (see Fig. 3 in^38^), but the positive effects of elevated [CO_2_] were still covered especially for the first time section (1998–2005/2006). Therefore, the ES of forbs (incl. legumes) is not a good indicator for the CFEs. In the following analysis, we will mainly focus on the reactions of the aboveground total biomass, as well as grass biomass to the emergence of ECEs. The results regarding forbs (incl. legumes) will be shown in the Supplementary Materials (Fig. S9).

**Table 1:** *P*-values for effect of the factors: Time, CO_2_ treatment, block, time × CO_2_, and time × block, on the biomass (Total, Grasses, and Forbs & Legumes) examined by repeated measures ANOVA.

PFG	Factors	H1			H2		
		1998–2005	2006–2013	1998–2013	1998–2005	2006–2013	1998–2013
Total	Time	0.033^*^	0.019^*^	0.015^*^	0.026^*^	0.099	0.051
	CO_2_	*n*.*s*.	0.108	*n*.*s*.	*n*.*s*.	0.007^**^	*n*.*s*.
	Block	0.047^*^	*n*.*s*.	*n*.*s*.	*n*.*s*.	0.054	*n*.*s*.
	Time × CO_2_	*n*.*s*.	*n*.*s*.	*n*.*s*.	*n*.*s*.	*n*.*s*.	*n*.*s*.
	Time × Block	*n*.*s*.	*n*.*s*.	*n*.*s*.	*n*.*s*.	*n*.*s*.	*n*.*s*.
Grasses	Time	0.026^*^	0.032^*^	0.006^**^	0.023^*^	0.013^*^	0.032^*^
	CO_2_	0.004^**^	0.053	0.005^**^	0.016^*^	*n*.*s*.	0.070
	Block	0.002^**^	0.032^*^	0.002^**^	0.047^*^	*n*.*s*.	*n*.*s*.
	Time × CO_2_	*n*.*s*.	*n*.*s*.	*n*.*s*.	*n*.*s*.	*n*.*s*.	*n*.*s*.
	Time × Block	*n*.*s*.	*n*.*s*.	*n*.*s*.	*n*.*s*.	0.039^*^	*n*.*s*.
Forbs & Legume	Time	0.016^*^	0.017^*^	0.019^*^	0.071	*n*.*s*.	0.039^*^
	CO_2_	*n*.*s*.	*n*.*s*.	*n*.*s*.	*n*.*s*.	*n*.*s*.	*n*.*s*.
	Block	*n*.*s*.	*n*.*s*.	*n*.*s*.	*n*.*s*.	*n*.*s*.	*n*.*s*.
	Time × CO_2_	*n*.*s*.	*n*.*s*.	*n*.*s*.	*n*.*s*.	*n*.*s*.	*n*.*s*.
	Time × Block	*n*.*s*.	*n*.*s*.	*n*.*s*.	*n*.*s*.	*n*.*s*.	*n*.*s*.

The effect was assessed for each harvest (H1 and H2) and the rmANOVA model was used at the full time series, as well as the two half time sections as suggested by^38^. For significant effect at *P* < 0.05, one asterisk was marked, while for *P* < 0.01, we use two asterisks. ‘n.s.’ indicated non-significant effect at *P* > 0.1.

**Figure 1:**
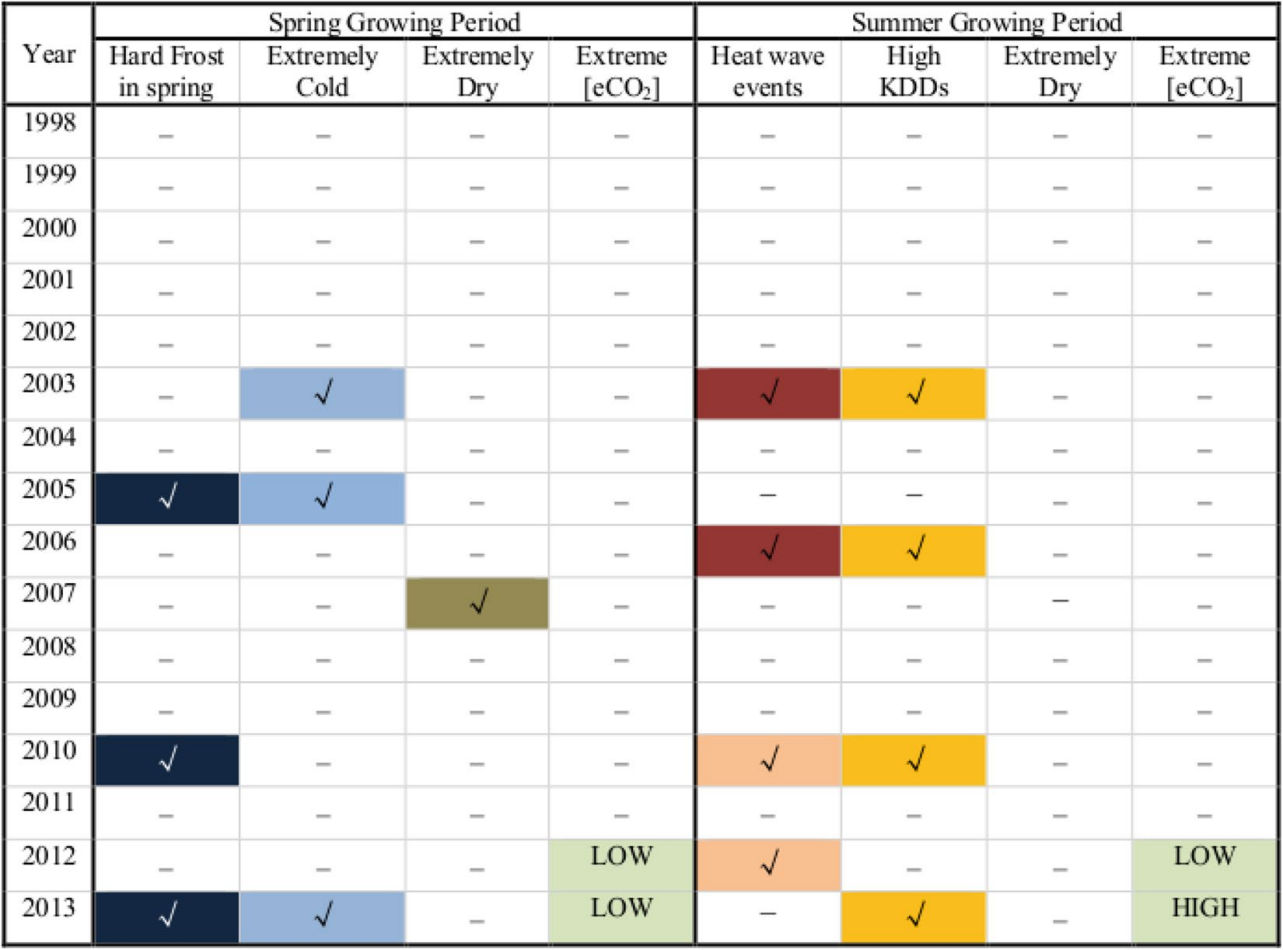
Detected extreme climate events (ECEs) in the spring and summer growing periods for years from 1998 to 2013. The colored boxes with check marks depict the occurrence of ECEs in the corresponding years. For the spring growing period, ECEs including hard frost in spring, extreme cold and dry events are shown, while for the summer growing period, heat wave events, high KDDs, and extreme dry events are detected. For the years that experienced a strong heat wave event, we used a dark red color. For the years that did not experience a strong heat wave event, but are very close to satisfying the conditions for being a heat wave event (4 days in a row with *T*_*max*_ > 30 °C), we used a light red color. Besides ECEs, extreme [CO_2_] enrichment events are also shown.

**Figure 2:**
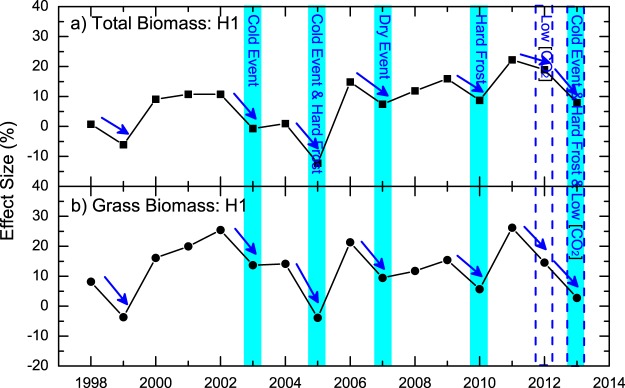
Connections between ECEs and the effect size (ES) of aboveground biomass in the spring harvest (H1). (**a**) shows the ES of the aboveground total biomass, (**b**) shows the ES of the aboveground grass biomass. The blue columns mark the years with ECEs, and the type of ECEs are shown within each column. For years with extreme [CO_2_] enrichment events, the columns are in dashed-borders. In both (**a**) and (**b**), the decreases of ES are marked by blue arrows. As one can see, for all the years with ECEs, the ES of both total biomass and grass biomass decreased.

**Figure 3:**
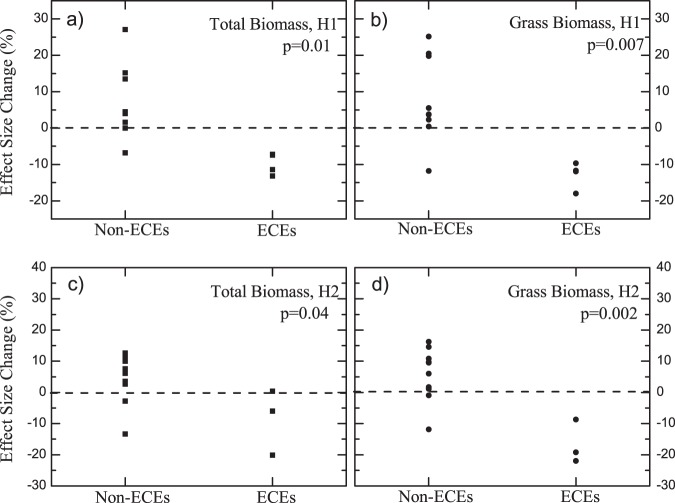
ES changes under different environmental conditions. The ES changes (compared to the previous year) were classified into two groups according to the occurrence of ECEs. (**a**), (**c**) show the results for the total biomass, for H1 and H2 respectively; while (**b**), (**d**) show the results for the grass biomass. The dashed line in each sub-figure is the zero line. The two groups in each figure are well separated. By applying student′s t-test (two sided), the differences are statistically significant with *p* = 0.01,0.007,0.04, and 0.002 for the sub-figures from (**a**–**d**), respectively.

Using various environmental and meteorological datasets (see “Material and Method” section), different ECEs were identified. For the spring growing periods, we identified extreme cold events in 2003, 2005, and 2013; spring hard frost events in 2005, 2010, and 2013; as well as extreme dry events in 2007^39^ (Fig. 1). For each year with ECEs, see Fig. 2a, the ES of total biomass in H1 was found to be lower than in the previous year, while for most non-extreme years, the ES in H1 was higher than in the previous year or remained unchanged. There were only two years (1999 and 2012) where lower ES values were found but not related to ECEs. For 1999, one explanation for the relatively low ES may be attributed to the initial unbalanced effects of the FACE experiment, as the Gi-FACE experiment started in 1998. For 2012, the low ES were most probably related to the extremely low [CO_2_] enrichment, which was caused by technical problems (Fig. S2). In fact, due to the low [CO_2_] enrichment in 2013, together with the extreme cold events, an even lower ES was found in 2013 than in 2012 (Fig. 2a), indicating a combined effect of low [CO_2_] enrichment and ECEs. Similar results were found for the grass biomass (Fig. 2b). If we remove the potential effects of extremely low [CO_2_] enrichment, and classify the ES changes (compared to the previous year) from 1999–2011 into two groups according to the occurrence of ECEs, the ES changes were well separated (Fig. 3a,b). For the years with ECEs, the ES decrease were statistically significant with *p* = 0.01 for the total biomass and *p* = 0.007 for the grass biomass. Therefore, the ECEs in spring played a major role in decreasing the ES.

**Figure 4:**
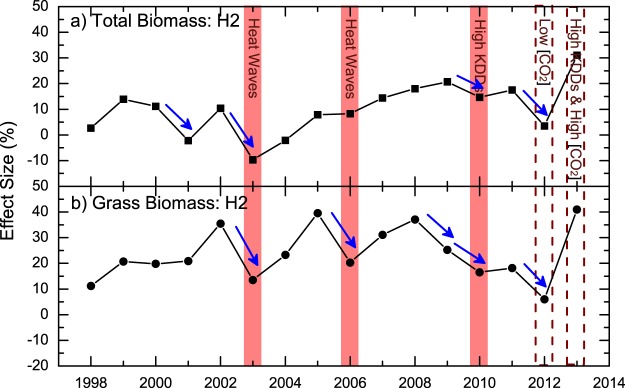
Connections between ECEs and ES of aboveground biomass in the summer harvest (H2). (**a**) shows the ES of the aboveground total biomass, (**b**) shows the ES of the aboveground grass biomass. Similar to Fig. 2, the ECEs are marked with red columns. For years with extreme [CO_2_] enrichment events, the columns have red dashed-borders. In both (**a**) and (**b**), the decreases of ES are marked by blue arrows. As one can see, for most years with ECEs, the ES of both total biomass and grass biomass decreased. Only one exception was found in 2006 for the total biomass.

For the summer growing period, prolonged heat wave events were detected in 2003 and 2006 (Fig. 1), with twelve and nine consecutive days with daily maximum temperature *T*_*max*_ higher than 30 °C, respectively (Fig. S5). By calculating the sum of maximum temperatures exceeding 30 °C (killing degree days [KDDs], see “Material and Method” section, similar to the definition in^40^), we found also high values in 2010 and 2013 (Fig. S4c), suggesting potential damages from high temperature in these years. Considering that there were four consecutive days with *T*_*max*_ > 30 °C in 2010 (Fig. S5), which could almost be classified as a heat wave event (see Fig. 1, the light red color), we determine that the summer growing period in 2010 also experienced extreme hot events. While for 2012 and 2013, extreme low and high [CO_2_] enrichments were observed, respectively (Figs 1 and S2). Therefore, to remove the potential side effects from the extreme [CO_2_] enrichments, we only consider the ECEs in 2003, 2006, and 2010. For the total biomass in H2, ES decreases were found in two of the three years (2003, 2010) (Fig. 4a). Only in 2006, the ES was not decreased, which was most probably due to the sudden increase of ES of forbs (Fig. S9b). After removing the forbs from the total biomass, ES decreases were observed in all the three years (Fig. 4b). Conversely, for the majority of the other years without ECEs, the ES was higher than in the previous year or remained unchanged (Fig. 4). If we classify the ES changes (compare to the previous year) into two groups according to the occurrence of ECEs, clear separations were again revealed (Fig. 3c,d). For the years with ECEs, the ES decreases are statistically significant with *p* = 0.04 for the total biomass and *p* = 0.002 for the grass biomass. Therefore, the CFEs in H2 also decreased significantly under the effects of ECEs.

To confirm these findings, we also compared the different CFEs using a modification of the new method proposed by Obermeier *et al*.^35^, who quantified CFEs as the slopes of productivity versus actual measured [CO_2_] to cope with the varying CO_2_ concentrations in real-world experiments. Fig. S10 shows the case in 2002 versus 2003 (H1, H2) and the case in 2009 versus 2010 (H1, H2) for total biomass, while Fig. S11 shows the results for grass biomass. For the years without ECEs (2002 and 2009), stronger responses of the total productivity to increased [CO_2_] were found (Fig. S10). While for years with ECEs (2003 and 2010), the slope decreased, vanished, or even became negative, suggesting smaller CFEs under the stress of ECEs. Similar results were obtained for the grass biomass (Fig. S11). Here, it should be denoted, that the amounts of the biomass harvested from H1 and H2 were at different levels (around $300 \sim 500g/{m}^{2}$ and $200 \sim 350g/{m}^{2}$ for H1 and H2, respectively, compare Figs S10a and S10b), and thus the linear regression method could not be applied for H1 and H2 together. Therefore, the comparison of harvests from two adjacent years as was done here resulted in only six observations which constrained the power of the statistical data analysis. Consequently and in combination with the large fluctuations, the differences of the slopes were not statistically significant. To compensate for this limitations, we additionally defined a “standardized” index, which allowed to include the data points from both harvests (shown in Fig. S12). Here, statistically significant and pronounced differences between the CFEs in years without ECEs (2002 and 2009) compared to years with ECEs (2003 and 2010) were observed.

## Discussion and Conclusion

In this study, we investigated the combined effects of extreme climatic events (e.g. heat wave events, extremely hot/cold events, extremely dry events, as well as hard frost events, etc.) and elevated [CO_2_] on aboveground biomass production. Beyond the recent work by Obermeier *et al*.^35^ where only the summer growing season data were considered, we focused also the spring growing season and ECEs such as the frost event were also studied. Rather than estimating impacts from the changes of mean climate conditions, we analyzed the effects of single extreme events, which may better reveal the response of biotic system to climate drivers. For both spring and summer, we found down-regulated effect size of aboveground biomass when extreme climatic events occurred during the growing season. The strongest decreases were associated with intensive and prolonged heat waves. In contrast to previous theories that suggest that stronger CO_2_ fertilization effects may be expected under higher temperatures and drier conditions^3,18–20^, our results suggest the CO_2_ fertilization effects can be lower if the growth conditions are too harsh, e.g., when heat wave events and droughts occur. This is reasonable as plant growth is influenced by multiple factors. Besides water, CO_2_, and light, plants also depend on factors including nutrient availability, temperature, pathogens, and herbivores. Stress from ECEs may limit plant growth via reduced enzyme activity, increased vulnerability to pathogens and herbivores, increased respiratory losses, etc. Accordingly, the high availability of CO_2_ cannot be fully utilized by plants. Our results are different to previous theories, but do not violate them. We argue that the previous theories^3,18–20^ are only applicable within a certain range, which may be determined by the local average environmental conditions^35,41^. Exceeding this optimal range, e.g., under extreme climatic events, the CO_2_ fertilization will no longer overrule the plant growth.

Besides the growing periods with ECEs, there are also few cases (e.g., 1999 and 2012 for both total biomass and grass biomass in H1; 2009 and 2012 for grass biomass in H2) where the decreased ES were not related to ECEs. Accordingly, the occurrence of ECEs is only a sufficient condition for the decrease of ES, not a prerequisite. The changes of ES can be influenced by other factors such as anomalous low [CO_2_] enrichment, or PFG competitions. For instance, the decreased ES in both harvest of 2012 were most probably related to the extremely low [CO_2_] enrichment (Fig. S2), which was caused by technical problems. For H2 in 2009, the competition between grass and other plant functional types (forbs, legumes) may have contributed to the decreased ES of grass biomass (Fig. S13). The interactions between plants also plays an important role, e.g., in dry growing periods, one of the dominant grasses and biomass builder (Arrhenaterum elatius) reduces significantly its growth but is only in parts replaced by other species. This was part of other studies^38^. In our analysis, only the abiotic climatic factors were considered, biotic factors and species interactions were disregarded.

**Figure 5:**
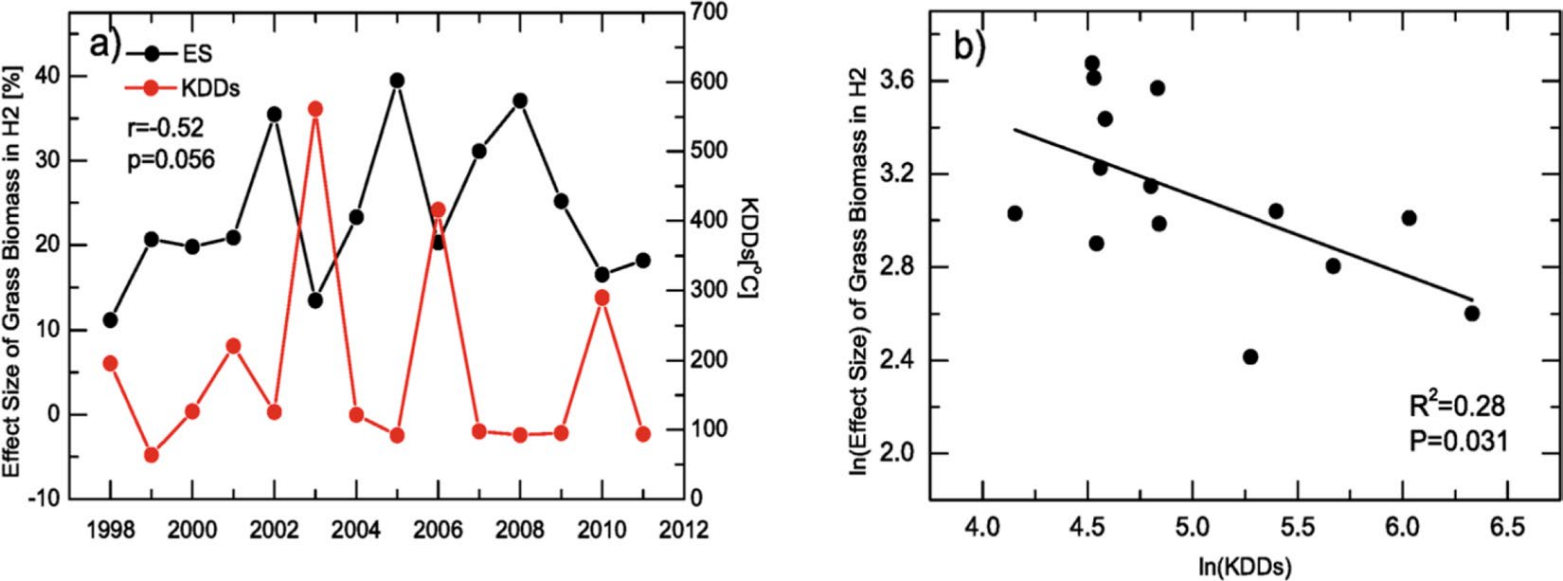
Relationships between the killing degree days (KDDs) and the effect size (ES) of grass biomass in the summer harvest (H2). To remove the side effects of extreme [CO_2_] enrichment events in 2012 and 2013, only the early 14 years data (1998–2011) were used for this figure. (**a**) Time series of KDDs (red curve, refer to right axis) and the ES (black curve, refer to left axis). (**b**) Regression analysis, 28% variance of the natural logarithm effect size can be explained by the natural logarithm KDDs, which is significant with *p* = 0.031. For the results without logarithmic transformation, please refer to Fig. S14.

In view of the non-negligible effect of ECEs, it is necessary to include the effects of ECEs for the understanding of ecosystem responses to increased [CO_2_]. Properly quantified indexes that present the effects of ECEs may be important for future projections. In our work, we calculated the killing degree days (KDDs) as one measure of the extreme events. Actually, it can also serve as an useful indicator of the negative effects of high temperatures in summer. Before showing the relations between KDDs and ES in H2, it is worth noting that the enrichment level of [CO_2_] was set as 20% above the ambient level and in most years, the actual measured enrichment level was indeed around 20% (Fig. S2). However, due to technical problems, the [CO_2_] enrichment in summer growing period was extremely low (7.62%) in 2012, and extremely high (48.53%) in 2013 (see Fig. S2 and Table S5). As a result, the ES in H2 may be altered largely due to the extreme low (high) enrichment levels in 2012 (2013). To avoid obtaining biased results from the damaged treatment design, we study the relations between ES and KDDs using the early 14-year data (1998–2011). As shown in Fig. 5, a significant negative correlation between KDDs and ES of grass biomass was found. For high KDDs, the ES dropped, while when the KDDs were low, the effect size increased. we calculated the correlation between ES and KDDs, which yielded a coefficient r of −0.52 with p = 0.056. The regression analysis indicated that 28% of the variance in the natural logarithm ES could be explained by the natural logarithm KDDs, which was significant with p = 0.031 (Fig. 5b; see S14 for the same result but without natural logarithm transformation). Therefore, we have reason to believe the extremely high temperatures may control the ES of the grass biomass during summer, which may be associated with the reduced transpiration caused by reduced stomatal conductance^42^ and increased respiration^23^. In this case, KDDs could potentially be used for development as an index for future projections of the response of the grassland to increased [CO_2_].

Our work focused on a grassland in central Europe. For other ecosystems, although different ECEs may occur and play different roles, we believe that the findings should be similar, in that (i) the ECEs can down-regulate the CFEs, and (ii) a properly quantified index (e.g. KDDs) may help explain the changes in CFEs. As extreme climatic events such as drought, heat waves, etc., are projected to increase in both frequency and intensity^14,15^, the mitigatory effects of C3 grassland ecosystems are likely to be restricted, leading to an accelerated warming trend in the future. To better understand atmosphere-vegetation interactions and further alleviate the uncertainties of future projections, additional results from other long-term studies over different climate zones are required.

## Materials and Methods

### Site description

The Gi-FACE experiment was carried out at a field site with an area of 1.5 ha in the German federal state of Hesse, near the town Giessen (50°32′N, 8°41′E) at 172 m a.s.l. The local annual mean precipitation over the research period was 558 ± 92 mm and the annual mean 2m-air temperature was 9.4 ± 0.1 °C. The research area has been managed as a meadow. It was mowed twice a year and not ploughed for at least 100 years. The old, non-grazed grassland has been fertilized with 50–80 kg N ha^−1^ yr^−1^ up to 1995. Afterwards nitrogen fertilization was reduced to 40 kg N ha^−1^ yr^−1^. The harvested biomass of this species rich grassland is dominated by grass, with small amounts of forb and legume included. The FACE experiment started in May 1998 and the mean [CO_2_] enrichment is +20 % above ambient during daylight hours. There are three circular plots (rings) subjected to elevated [CO_2_] (eCO_2_), while another three circular plots (rings) served as controls with ambient [CO_2_] (aCO_2_) (see Fig. S1). They were arranged in a randomized block design (three blocks). Each ring had an inner diameter of 8 m with an inner circular buffer-zone of 0.9 m. Biomass was harvested twice a year before the end of spring (H1) and summer (H2). In each harvest from 1998 on, the vegetation was cut manually with garden scissors at 3–5 cm above the soil surface. The harvested aboveground biomass was stored at 4 °C and sorted by hand into three functional groups: grasses, forbs and legumes. For more details of the site, please refer to^37,38^.

### Data description

Aboveground biomass harvested from both spring (H1) and summer (H2) were used in this study. The spring harvest date was around the end of May (beginning of June) each year, while the summer harvest date was around the beginning of September (see Table S3). Mean biomass calculated over the three elevated [CO_2_] plots and biomass averaged over the three ambient [CO_2_] plots were used to quantitatively show the CO_2_ fertilization effects. Besides biomass, daily soil moisture (volumetric water content in 10 cm soil depth, averaged from the measurements of 4 probes in each plot), semi-hourly 2m-air temperature, semi-hourly precipitation, as well as the hourly mean of CO_2_ concentration measured in the center of each ring, were used in this study. The soil moisture (averaged over the six rings) and precipitation are used for the determination of extreme dry events, while the 2m-air temperature records are used for the determination of extreme cold/hot events (as well as hard frost events and heat wave events), and also the calculation of Killing Degree Days (KDDs). With the measured CO_2_ concentration in each ring, we investigated the true [CO_2_] enrichment (see Fig. S2). Before determining extreme events, new data such as the number of days with daily maximum temperature higher than 30 °C, the number of consecutive rain free days, the averaged daily minimum temperatures, etc., were derived for each growing period, to better show the environmental properties (see Tables S4 and S5).

### Statistical Analysis

#### Determination of the growing periods

For spring, the start of growing season was defined as the first day after winter, when the daily mean air temperature is higher than 5 °C (Table S4), as phenological observations showed significant aboveground growth from that day onwards and CO_2_ flux measurements show a net CO_2_ assimilation. The end of growing season thus was the harvest day of H1 (Table S3). For summer, the start of growing season was the first day after H1, while the end of growing season was the harvest day of H2 (around the beginning of September).

#### Calculation of Effect Size (ES)

The CO_2_ fertilization effects in this study are represented by the effect size (ES) of aboveground biomass, which is defined as:

1 $$ES=\frac{Bio(eC{O}_{2})-Bio(aC{O}_{2})}{Bio(aC{O}_{2})}\times \mathrm{100 \% ,}$$

where *Bio*(*eCO*_2_) stands for the dry biomass matter obtained from eCO_2_ plots, while *Bio*(*aCO*_2_) represents the dry biomass matter obtained from the aCO_2_ plots. It is worth to note that there are three blocks in the experiment, and each block consist of two plots (one eCO_2_ plots and one aCO_2_ plots). Although the plots within each block are closely located (Fig. S1), they may still carry different background information which could further affect the productivity. Besides, from the rmANOVA (Table 1), the factor “Block” has significant effects for both the total biomass and the grass biomass, indicating inter-replicate discrepancies. To remove this background information, we used the averaged biomass (over the 3 replicates) to better estimate the CO_2_ fertilization effects.

#### Repeated-measures ANOVA (rmANOVA)

To check whether the effect size (ES) was indeed induced by the elevated [CO_2_], the treatment effect was assessed by rmANOVA. Aboveground total biomass, grass biomass, and the forbs (incl. legumes) measured from the six plots (three blocks) were analyzed, respectively. Factors including time, CO_2_ treatment, block, as well as the interactions time × CO_2_, time × block were considered in the model. For each harvest (H1 and H2), the rmANOVA model was run for the full time period of 1998–2013. Meanwhile, as suggeted by the break point analysis in^38^, two half time sections, 1998–2005 and 2006–2013, were also analyzed by rmANOVA. If the *p*-values for the CO_2_ treatment is smaller than 0.05, we consider there were significant treatment effect and ES can be used to represent the CO_2_ fertilization effect.

#### Competition of Plant Functional Groups (PFGs)

To quantify the competition of grass with other plant functional groups (forb + legume), the Relative Changes (RCs) of grass percentage compared with that in the previous year were calculated (Table S1, S2). To quantitatively test for the different RCs in eCO_2_ rings and in aCO_2_ rings, the product of RC in eCO_2_ rings and RC in aCO_2_ rings were calculated for each year (Fig. S13). Positive products indicate that the grass percentages in eCO_2_ rings and in aCO_2_ rings changed towards the same direction (increased or decreased) compared with the previous year, while negative products depict different changing directions (increase and decrease). By definition, increased grass percentage in eCO_2_ rings and decreased grass percentage in a CO_2_ rings may contribute to an increasing ES of grass biomass (e.g., H1 in 2011), while a decreased grass percentage in eCO_2_ rings associated with an increased grass percentage in aCO_2_ rings lead to a decreasing ES of grass biomass (e.g., H2 in 2009).

#### Definition of Extreme Climatic Events (ECEs)

We have defined different ECEs, including extreme cold and hot events, extreme dry events, hard frost in spring, as well as heat wave events. Their definitions are shown below. It is worth to note that (i) two times standard deviation (2 SD) was widely used for the determination of ECEs, but other threshold (e.g. 1.5 SD) has also been checked, which gives robust results; (ii) when studying the period-averaged (accumulated) temperature (precipitation), linear trends over 1997–2013 were removed before the analysis.

Extreme cold events: For a given growing period, if (i) the minimum temperature averaged over this period was exceptionally low (exceeds 2 SD, based on the data from 1997–2013); or (ii) the number of days with below-zero daily air mean temperature (*T*_*mean*_ < 0 °C) was exceptionally high (exceeds 2 SD, based on the data from 1997–2013); or (iii) the consecutive days with *T*_*mean*_ < 0 °C was exceptionally long (exceeds 2 SD, based on the data from 1997–2013), we defined this growing period had experienced an extreme cold event (Fig. S3).

Extreme hot events: For a given growing period, if (i) the maximum temperature averaged over this period was exceptionally high (exceeds 2 SD, based on the data from 1997–2013); or (ii) the number of days with *T*_*max*_ > 30 °C was exceptionally high (exceeds 2 SD, based on the data from 1997–2013); or (iii) the consecutive days with *T*_*max*_ > 30 °C was longer than 5 days, we considered this growing period had experienced an extreme hot event (Figs S4, S5). Especially, when the condition (iii) was satisfied, we define a heat wave event (Fig. S5). The threshold 30 °C was determined according to the 95th percentile of the daily maximum temperature distribution, a definition similar to those used previously^43^.

Extreme dry events: For a given growing period, if (i) the precipitation accumulated over this period was exceptionally low (exceeds 2 SD, based on the data from 1997–2013); or (ii) the soil moisture averaged over this period was exceptionally low (exceeds 2 SD, based on the data from 1997–2013); or (iii) the consecutive rain free days were long enough to exceed 2 SD, we say this growing period had experienced an extreme dry event (Figs S6, S7).

Hard frost in spring: If in spring (March, April, and May) after the first day of the growing season, the daily minimum temperature dropped below −10 °C, we defined it as a hard frost event in spring (Fig. S8), which is believed to cause severe damages to vegetation^36^.

#### Calculation of Killing Degree Days (KDDs)

KDDs is the sum of maximum temperatures in excess of 30 °C, as shown below,

2 $$KDDs=\sum _{i=1}^{n}\,a{T}_{i},a=(\begin{array}{cc}1 & {T}_{i}\ge {30}^{\circ }C\\ 0 & {T}_{i} < {30}^{\circ }C\end{array}$$

where *T*_*i*_, *i* = 1,2, …, *n* represent the daily maximum temperatures from the beginning to the end of the growing period. KDDs is one indicator that represent the negative impacts of high temperature. Different from^40^, where the threshold was 29 °C, here in this study we use 30 °C, as this is the threshold we used to determine heat wave events. Fig. S4c shows the KDDs calculated for each year.

## Electronic supplementary material

Supplementary Material
