# Supplementary material for: Extreme climatic events down-regulate the grassland biomass response to elevated carbon dioxide
Source: Sci Rep. 2018 Dec 10;8:17758. doi: 10.1038/s41598-018-36157-x (PMC6288116; doi:10.1038/s41598-018-36157-x)
Supplement: Supplementary file 1 — Supplementary Material [file 41598_2018_36157_MOESM1_ESM.pdf]

Supplementary Material for

Extreme climatic events down-regulate the grassland biomass response to elevated carbon dioxide

Naiming Yuan[1,2*], Gerald Moser[3], Christoph Mueller[3,4], Wolfgang A. Obermeier[5], Joerg Bendix[5], and Jürg Luterbacher[1,6]

1 Department of Geography, Climatology, Climate Dynamics and Climage Change, Justus-Liebig University Giessen, Senckenbergstr. 1, 35390 Giessen, Germany

2 CAS Key laboratory of Regional Climate-Environment for Temperate East Asia, Institute of Atmospheric Physics, Chinese Academy of Sciences, Beijing, 100029, China

3 Department of Plant Ecology, Justus-Liebig University Giessen, Heinrich-Buff-Ring 26, 35392 Giessen, Germany

4 School of Biology and Environmental Sciences, University College Dublin, Ireland

5 Faculty of Geography, Laboratory for Climatology and Remote Sensing, Philipps-University of Marburg, Deutschhausstr. 10, Marburg, Germany

6 Centre for International Development and Environmental Research, Justus-Liebig University Giessen, 35390 Giessen, Germany

*Correspondence author: Naiming Yuan, Department of Geography, Justus-Liebig University Giessen, Giessen, Germany. Email: naimingyuan@hotmail.com; Now at: CAS Key laboratory of Reginal Climate-Environment for Temperate East Asia, Institute of Atmospheric Physics, Chinese Academy of Sciences, Beijing, 100029, China; Tel:+86-(0)10-82995111

This file includes:

- Fig. S1. Aerial photo of the Gi-FACE field site.
- Fig. S2. Measured [CO_2] enrichment for spring and summer growing periods before the respective harvest dates.
- Fig. S3. Determination of extreme cold events during spring growing period.
- Fig. S4. Determination of extreme hot events as well as killing degree days (KDDs) during summer growing period.
- Fig. S5. Determination of heat wave events during summer growing period.
- Fig. S6. Determination of extreme dry events during spring growing period.
- Fig. S7. Determination of extreme dry events during summer growing period.
- Fig. S8. Determination of hard frost events in spring.
- Fig. S9. Effect size (ES) of forbs (incl. legumes).
- Fig. S10. Regression analysis of the aboveground total biomass versus CO_2 concentrations for growing periods with/without ECEs.
- Fig. S11. Regression analysis of the aboveground grass biomass versus CO_2 concentrations for growing periods with/without ECEs.
- Fig. S12. Relative changes of biomass between the years 2002/2009 and the years 2003/2010.
- Fig. S13. Quantitative analysis of the different changing directions of grass percentage between eCO_2 rings and aCO_2 rings, for H1 (A) and H2 (B).
- Fig. S14. Relations between the effect size of grass biomass in H2 and the KDDs.
- Table S1. Grass Percentage measured in each year, for H1.

- Table S2. Grass Percentage measured in each year, for H2.
- Table S3. Harvest dates of the Gi-FACE experiment.
- Table S4. Data derived from the growing period of each year, for spring.
- Table S5. Data derived from the growing period of each year, for summer.

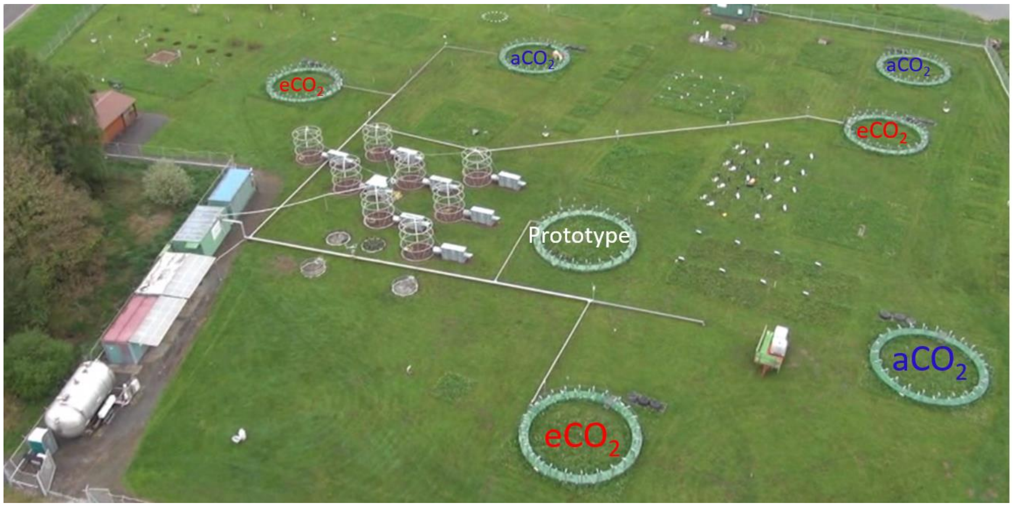

Fig. S1: Aerial photo of the Gi-FACE field site. Locations of the rings with elevated CO_2 (eCO_2) and ambient CO_2 (aCO_2) are indicated. Inner diameter of the rings is 8m. We acknowledge the Hessian Agency for Nature Conservation, Environment and Geology (HLNUG) for providing this photo.

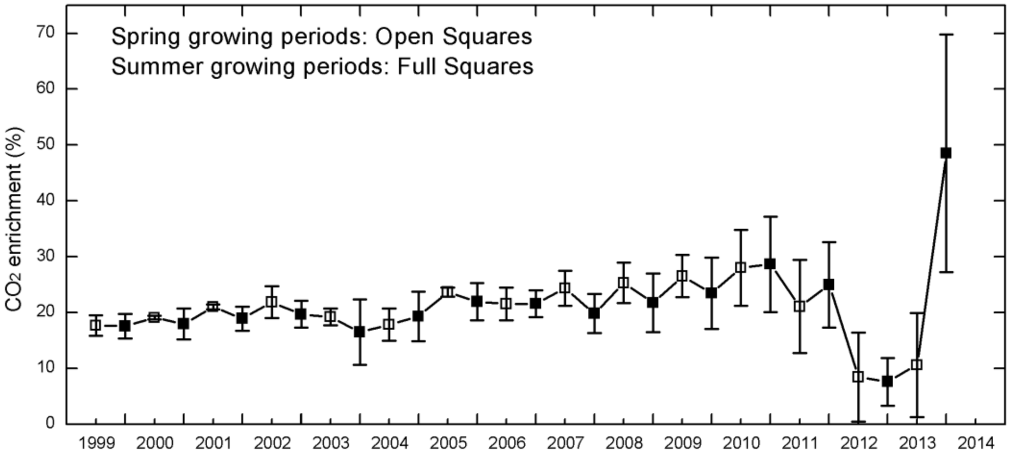

Fig. S2: Measured [CO_2] enrichment for spring and summer growing periods before the respective harvest dates. The open squares represent the [CO_2] enrichment of the spring Harvest (H1), while the solid squares stand for the [CO_2] enrichment of the summer Harvest (H2). The error bars are the one standard deviation calculated from the [CO_2] records over different rings. For H1 in 2012 and 2013, the [CO_2] enrichments were extremely low due to technical issues, while for H2 in 2012 and 2013, the [CO_2] enrichments were extremely low and high, respectively.

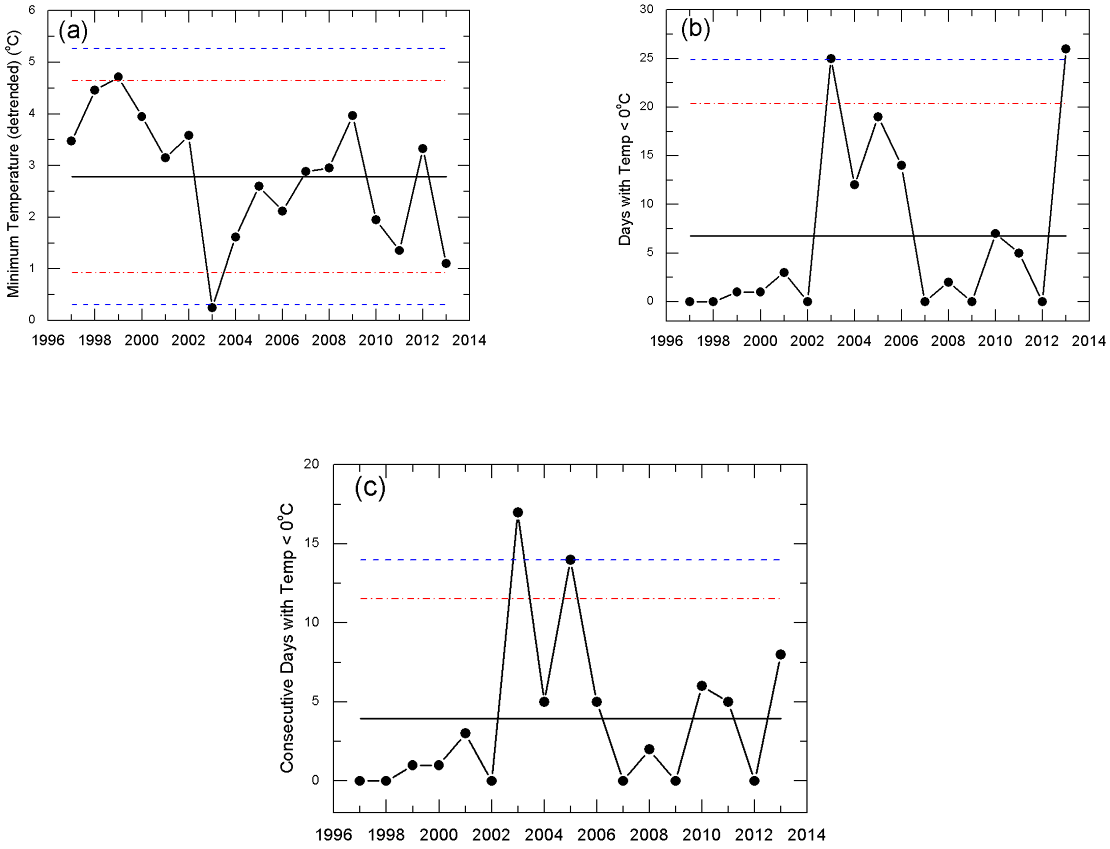

Fig. S3: Determination of extreme cold events during spring growing period. a) shows the daily minimum temperature (with linear trend over 1997-2013 removed) averaged over the growing period for each year, b) shows the number of days with Tmean<0°C during the growing period for each year, and c) shows the number of consecutive days with Tmean<0°C during the growing period for each year. The red and blue dashed lines represent the 1.5 and 2 times of standard deviations. Extreme cold events were found in 2003, 2005, and 2013.

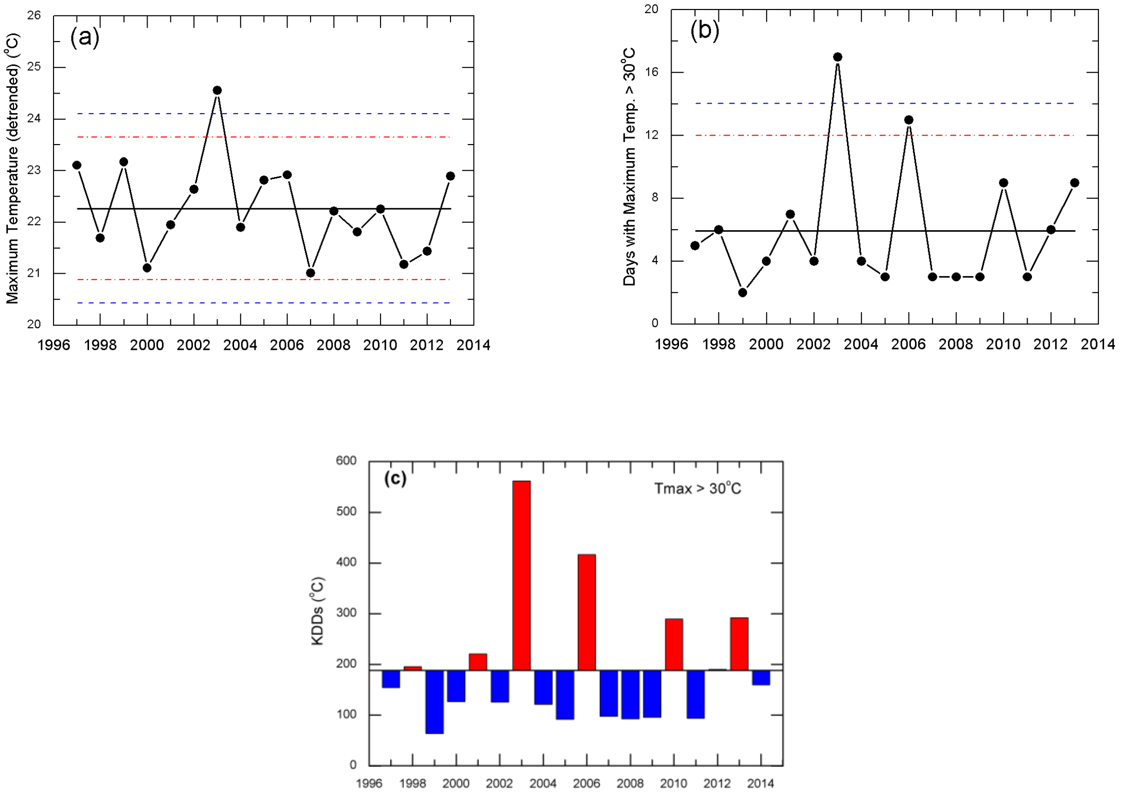

Fig. S4: Determination of extreme hot events as well as Killing Degree Days (KDDs) during summer growing period. a) shows the daily maximum temperature (with linear trend over 1997-2013 removed) averaged over the growing period for each year, b) shows the number of days with Tmax>30°C during the growing period for each year, and c) shows the KDDs calculated for each year. In 2003 the grassland had experienced an extreme hot event.

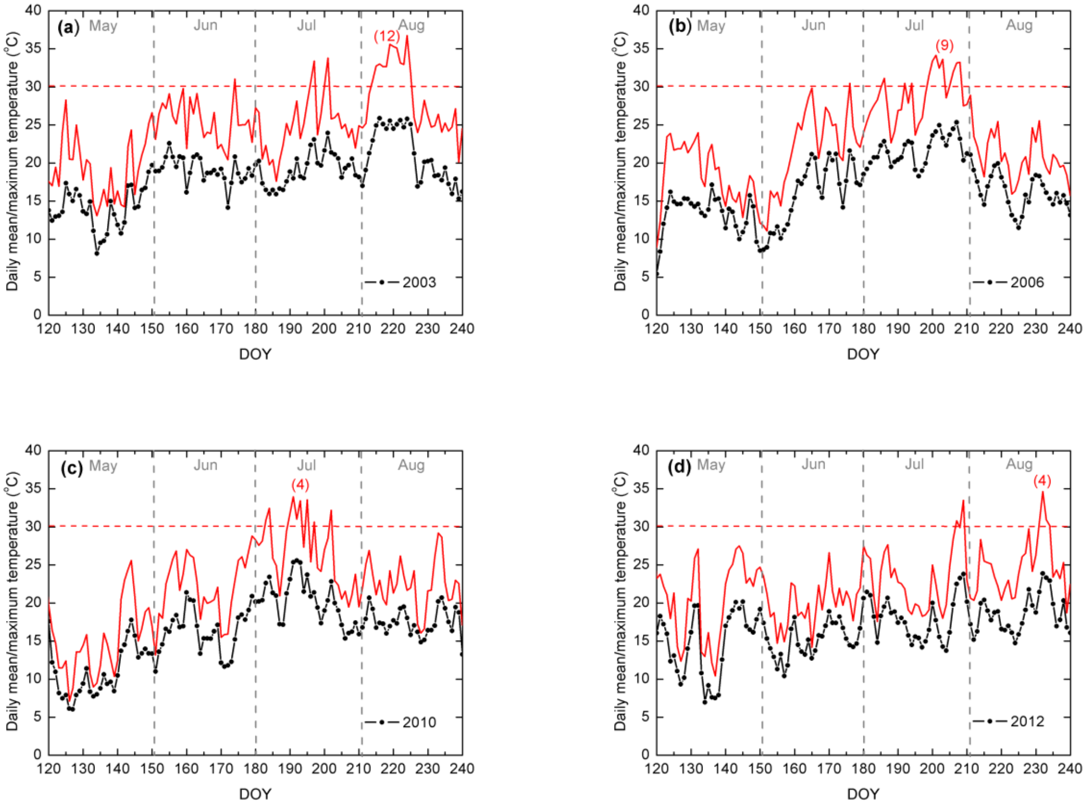

Fig. S5: Determination of heat wave events during summer growing period. The black curve represents the daily mean temperature, while the red curve shows the daily maximum temperature. The red number in each sub-figure represents the number of consecutive days with Tmax>30ºC. a) shows the results of 2003, while b), c), d) are for 2006, 2010, and 2012. There were strong heat wave events in 2003 and 2006.

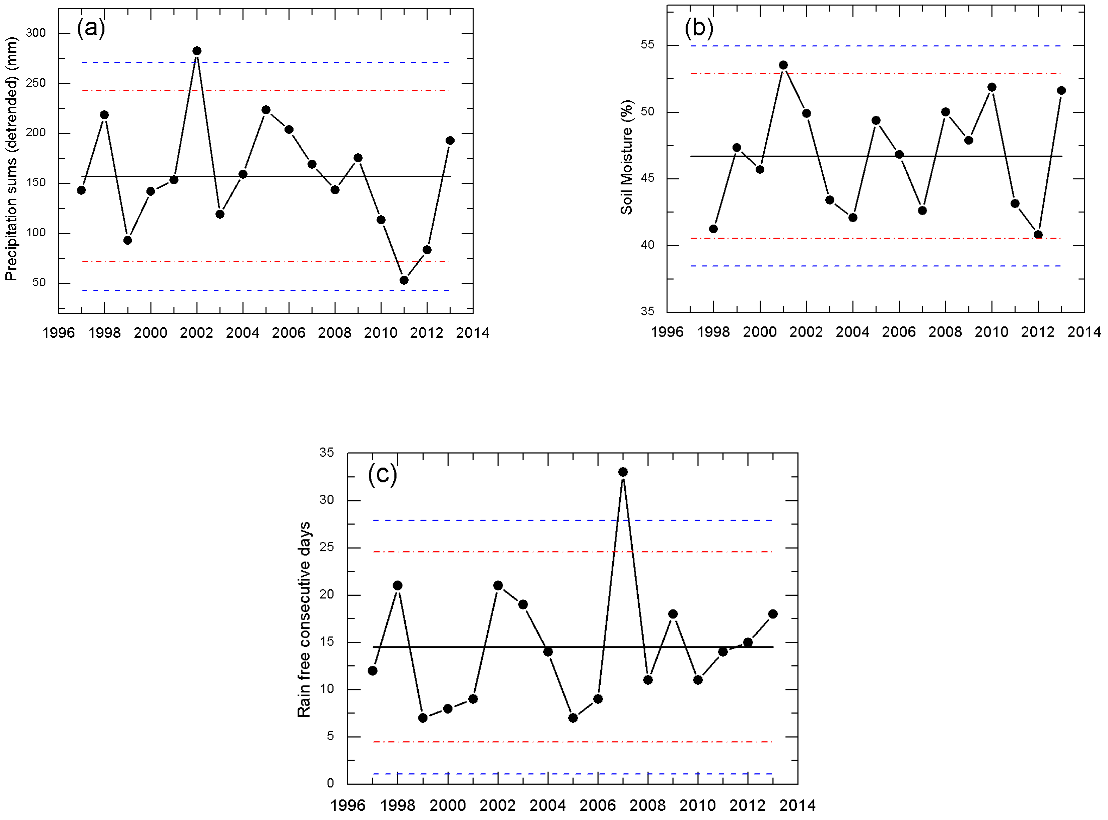

Fig. S6: Determination of extreme dry events during spring growing period. a) shows the precipitation (with linear trend over 1997-2013 removed) accumulated over the growing period for each year, b) shows the soil moisture averaged over the growing period for each year, and c) shows the number of consecutive rain free days during the growing period for each year. The red and blue dashed lines represents the 1.5 and 2 times of standard deviations. Extreme dry events occurred in 2007.

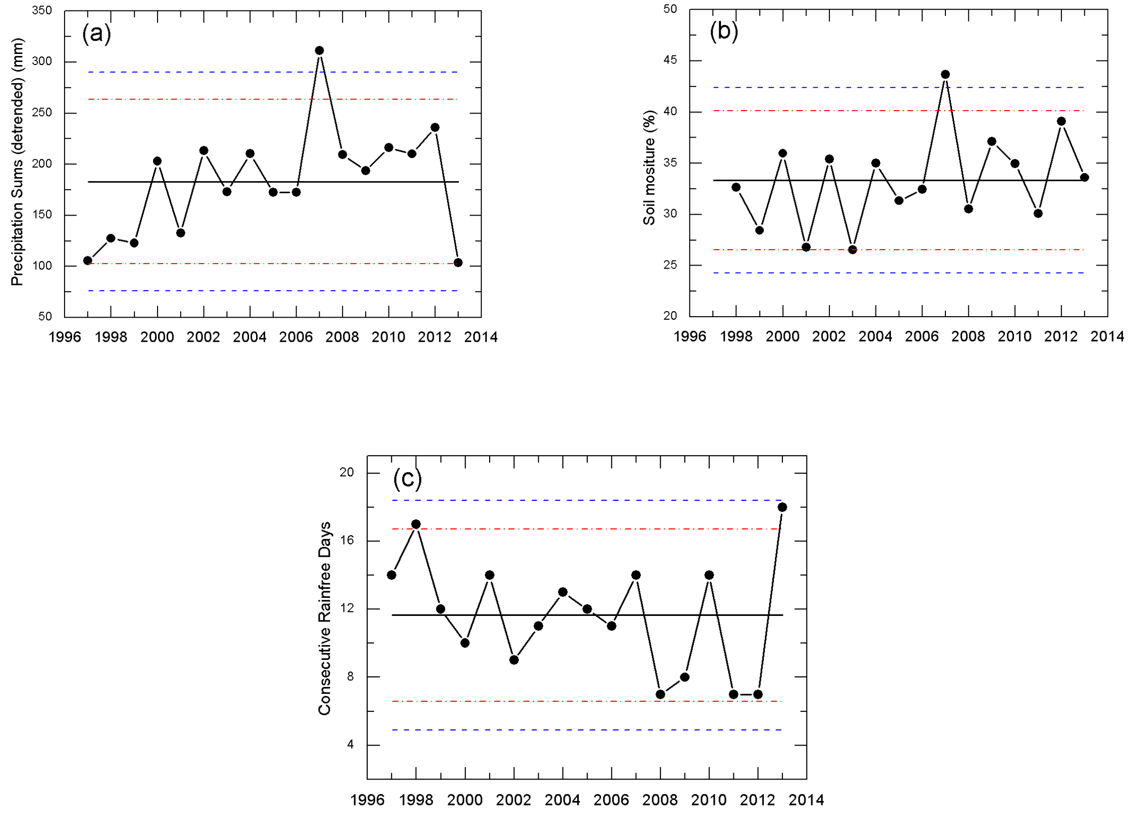

Fig. S7: Determination of extreme dry events during summer growing period. The same as Fig. S6, but for the summer growing period. There was no extreme dry event in the summer growing period, but an extremely wet season in 2007.

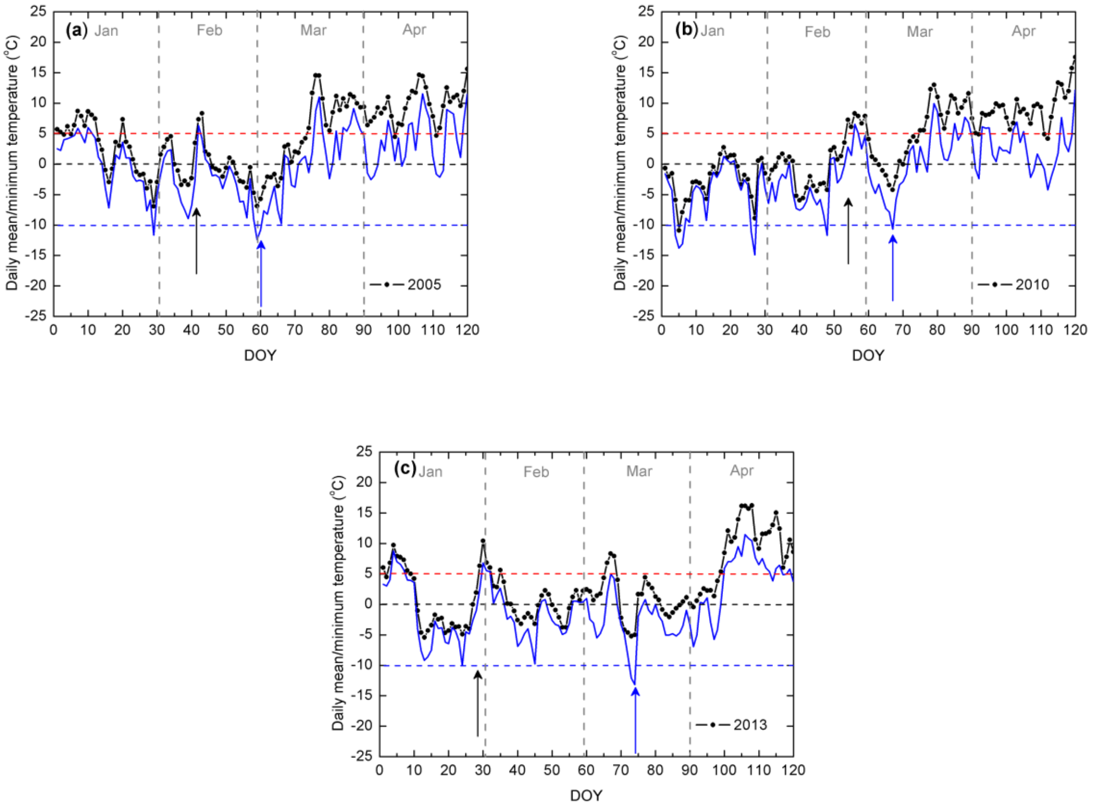

Fig. S8: Determination of hard frost events in spring. The black curve represents the daily mean air temperature, while the blue curve shows the daily minimum temperature. The black arrow points to the day when the growing season started and the blue arrow points to the day with hard frost event. a) shows the results of 2005, and b), c) are for 2010 and 2013. After the start of growing season, these three years all experienced hard frost events in spring.

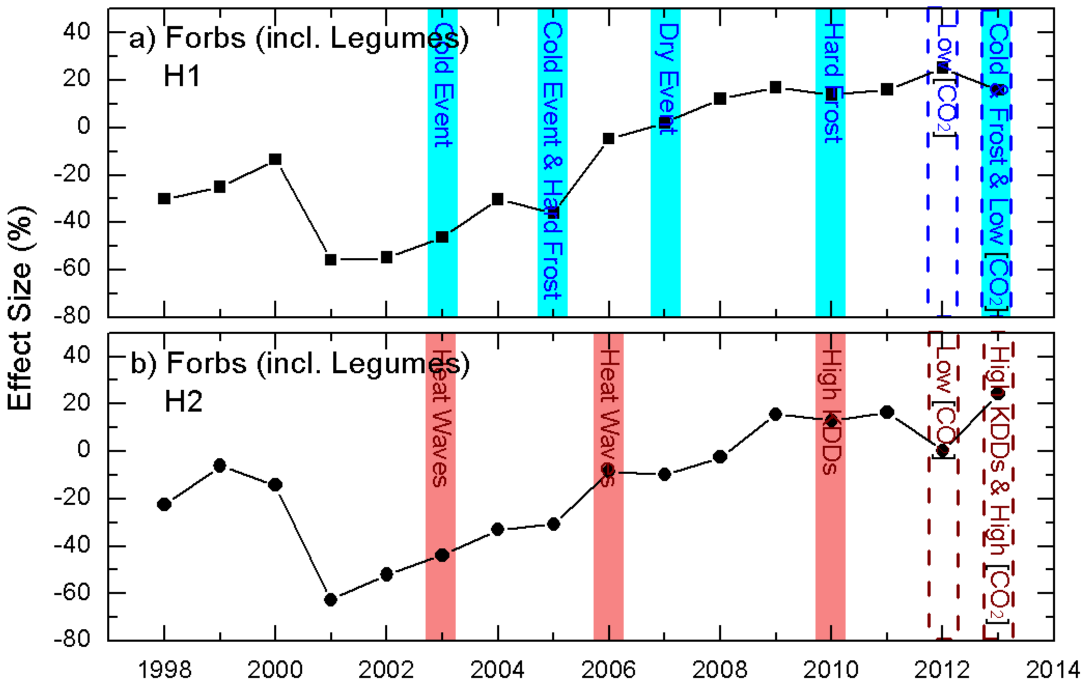

Fig. S9: Effect size (ES) of forbs (incl. legumes). a) shows the results for H1, b) shows the results for H2. The blue columns mark the years with ECEs in spring growing period, while the red columns mark the years with ECEs in summer growing period. The type of ECEs are also shown in each column.

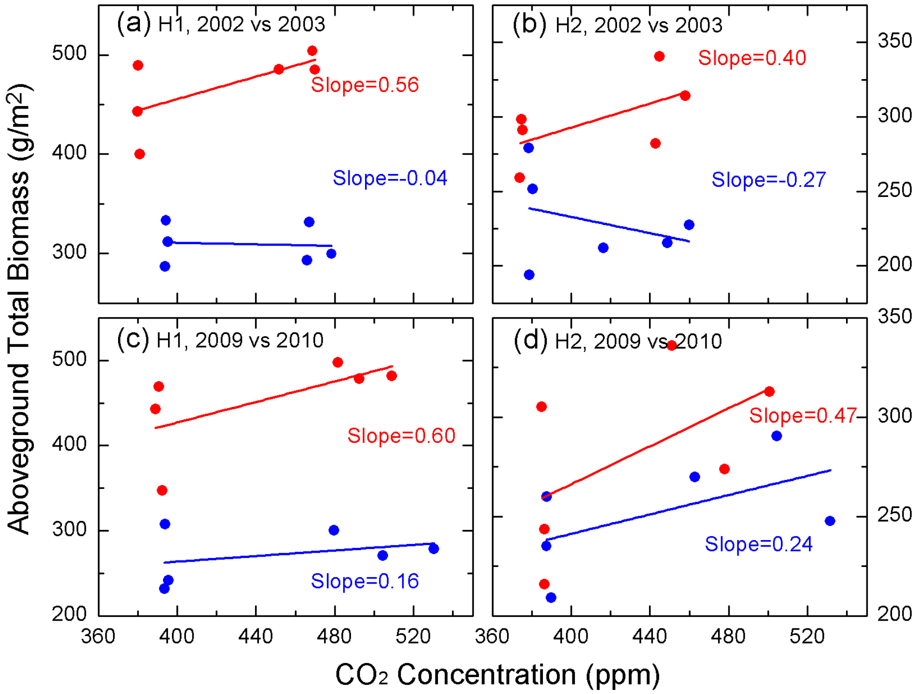

Fig. S10: Regression analysis of the aboveground total biomass versus CO_2 concentrations for growing periods with/without ECEs. a-b) indicate the results of 2002 (red) and 2003 (blue) for H1 and H2, respectively. c-d) show the results of 2009 (red) and 2010 (blue). For the growing periods without ECEs (2002 and 2009), steeper slopes are obtained indicating stronger links between biomass and CO_2 concentration. Conversely, for the growing periods with ECEs (2003 and 2010), the slopes are lower, indicating weaker CFEs. Although clear differences in the slopes in each sub-figure can be observed, it worth noting that the comparison of the two adjacent years is based on only six observations, which constrains the power of the statistical data analysis. Consequently and in combination with the large fluctuations, the differences of the slopes are not statistically significant, with p=0.13, 0.16, 0.31, and 0.57 for (a-d), respectively.

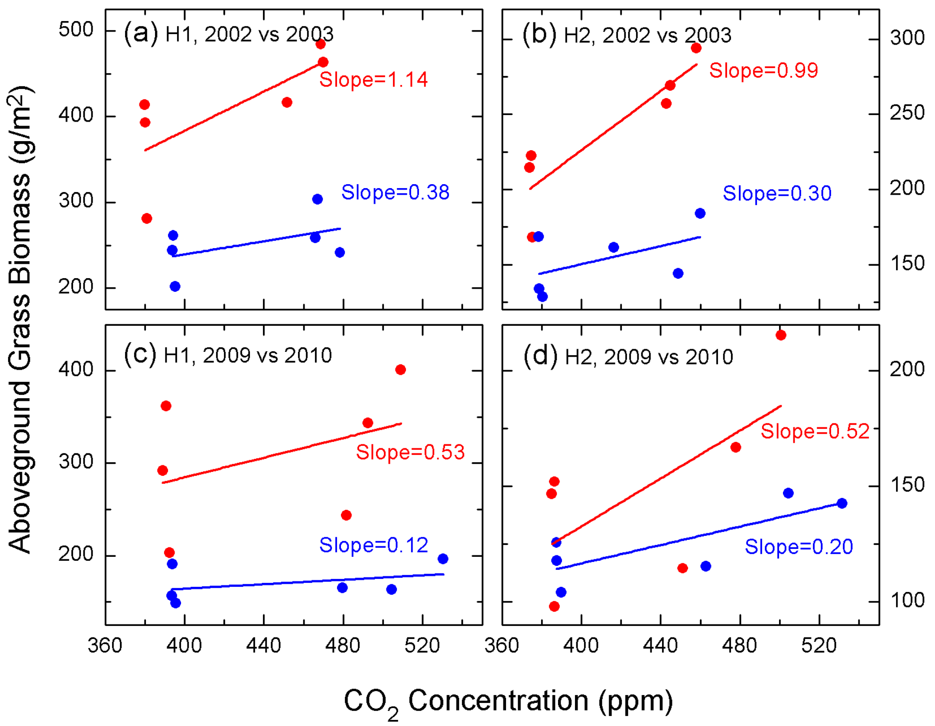

Fig. S11: Regression analysis of the aboveground grass biomass versus CO_2 concentrations for growing periods with/without ECEs. Similar to Fig. S10, but for the grass biomass. Although clear differences in the slopes in each sub-figure can be observed, constrained by the lack of data (only six observations) and the large fluctuations, the differences in the slopes in a), c) and d) are not statistically significant, with p=0.23, 0.50, and 0.31, respectively. Only in b), the difference is at the border of being significant, with p=0.05.

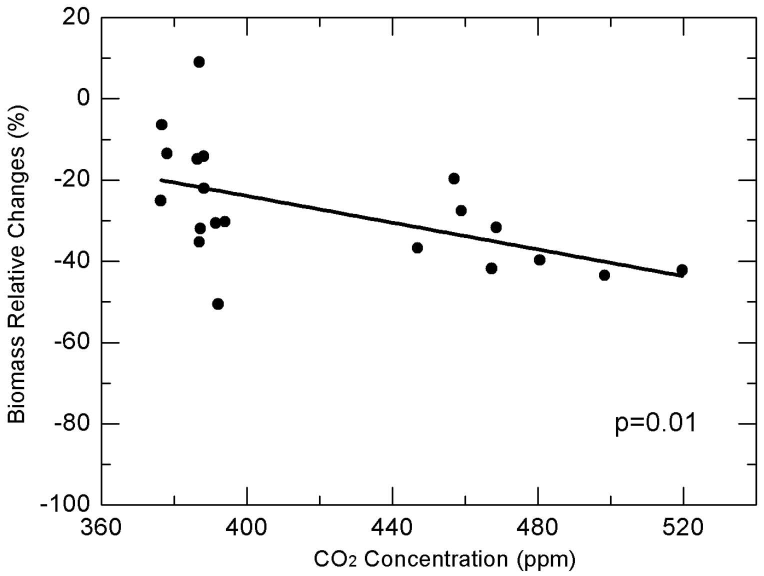

Fig. S12: Relative changes of biomass between the years 2002/2009 and the years 2003/2010. Using the biomass data shown in Fig. S10, relative changes of biomass (for each ring, each harvest) between the two adjacent years were calculated as "[(Biomass ϵ 2003/2010)-(biomass ϵ 2002/2009)] / (biomass ϵ 2002/2009)" (Y-axis). Taking the total biomass from H1 in 2002 and 2003 as an example, the relative changes were calculated as: for each ring, the biomass from H1 in 2003 first subtracted the biomass from H1 in 2002, then the difference was divided by the biomass from H1 in 2002. The same calculations were also done for H2, and for the years 2009 vs 2010. X-axis shows the corresponding [CO_2]. Since the actual measured [CO_2] varied over time, we only considered the cases when the differences of [CO_2] between the two adjacent years (2002 vs 2003, 2009 vs 2010) were smaller than 5%, and used the two-year averaged [CO_2]. Thus, 20 (out of 24) points were calculated for this figure. If the CO_2 fertilization effects (CFEs) in 2003/2010 indeed decreased compared to the CFEs in 2002/2009, the calculated biomass relative changes should decrease significantly with the increase of the [CO_2]. As expected, we observed this significant decreasing trend, with $p=0.01$.

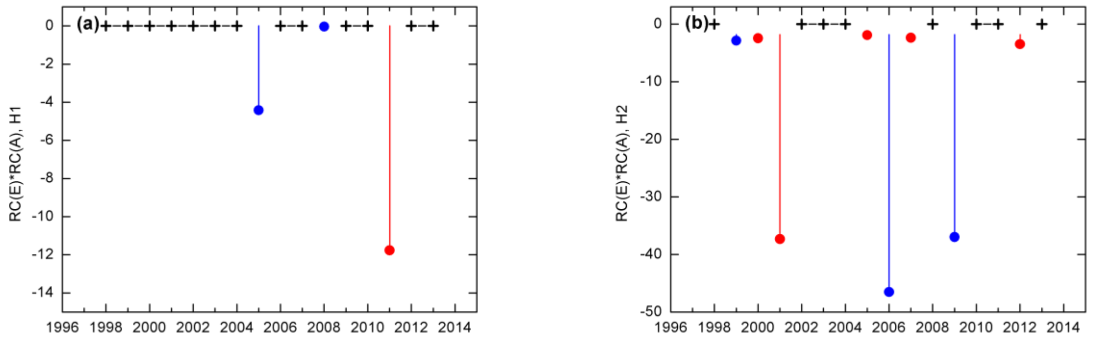

Fig. S13: Quantitative analysis of the different changing directions of grass percentage between eCO_2 rings and aCO_2 rings, for H1 (a) and H2 (b). Using the "Relative Change" (RC) values shown in Tab. S1 and Tab. S2, we calculated the product of RC in eCO_2 rings and RC in aCO_2 rings. Positive products indicate the same changing direction (increase or decrease) of grass percentage in eCO_2 rings and aCO_2 rings. In this case, we mark a plus (+). Negative products are shown with different colors. When RC in eCO_2 rings is positive but RC in aCO_2 rings is negative, we use red color. On the contrary, when RC in eCO_2 rings is negative but RC in aCO_2 rings is positive, we use blue color.

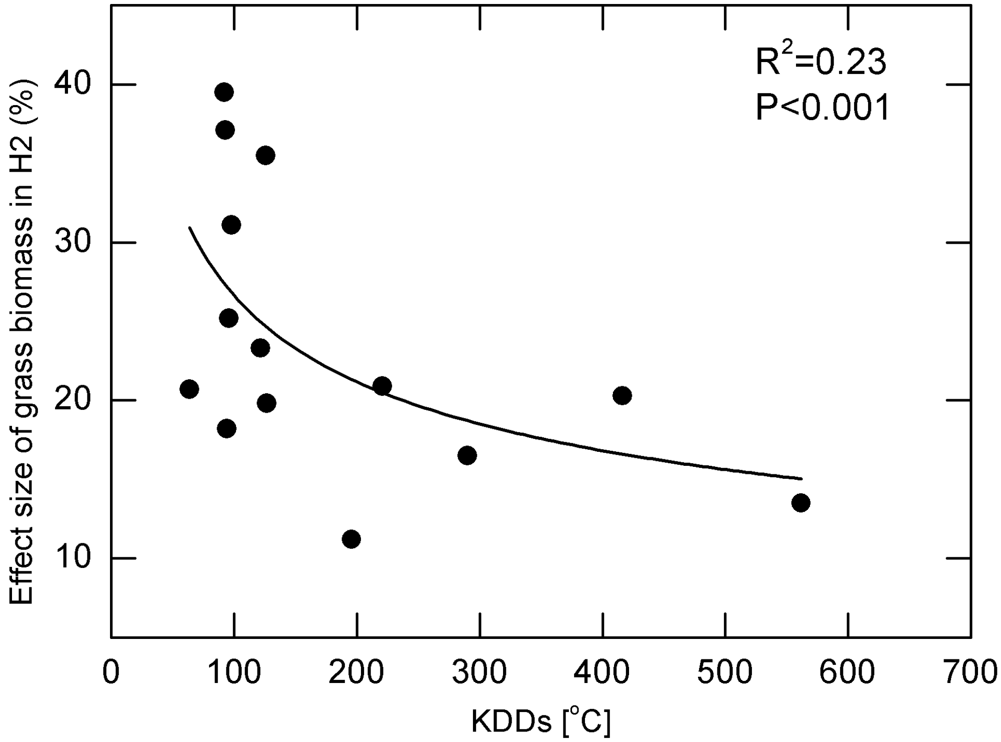

Fig. S14: Relations between the effect size of grass biomass in H2 and the KDDs. This figure is similar to the Fig. 5 in the main text, but the data are not transformed by natural log. By making power-law fitting, one can find significant relations ($p<0.001$) between the effect size of grass biomass and the KDDs.

Table S1: Grass Percentage measured in each year, for H1. In this table, the grass percentage (%) measured in H1 are shown in the 2nd column (for the eCO_2 rings, E rings) and the 4th column (for the aCO_2 rings, A rings). The numbers in the 3rd and the 5th columns are the relative changes (RC) of the grass percentage compared to that in the previous year.

Year (H1)	Grass Percentage in E rings (%)	Relative Change in E rings (%)	Grass Percentage in A rings (%)	Relative Change in A rings (%)
1997	88.3	-	83.8	-
1998	86.3	-2.22	80.8	-3.62
1999	90.3	4.63	88.1	9.02
2000	81.1	-10.16	76.4	-13.28
2001	95.2	17.37	87.9	15.08
2002	92.4	-2.94	81.3	-7.55
2003	86.7	-6.15	76.1	-6.39
2004	79.4	-8.50	69.7	-8.34
2005	78.8	-0.76	73.8	5.79
2006	79.6	1.12	74.6	1.14
2007	74.5	-6.46	72.2	-3.25
2008	74.4	-0.81	72.3	0.21
2009	68.0	-8.57	67.2	-7.12
2010	62.0	-8.85	63.7	-5.14
2011	63.4	2.29	60.5	-5.13
2012	57.0	-10.11	59.1	-2.34
2013	57.3	0.56	60.4	2.26

Table S2: Grass Percentage measured in each year, for H2. In this table, the grass percentage (%) measured in H2 are shown in the 2nd column (for the eCO_2 rings, E rings) and the 4th column (for the aCO_2 rings, A rings). The numbers in the 3rd and the 5th columns are the relative changes (RC) of the grass percentage compared to that in the previous year.

Year (H2)	Grass Percentage in E rings (%)	Relative Change in E rings (%)	Grass Percentage in A rings (%)	Relative Change in A rings (%)
1997	77.4	-	66.6	-
1998	80.4	3.85	74.6	11.99
1999	78.9	-1.86	75.0	0.59
2000	80.3	1.84	74.7	-0.36
2001	89.5	11.34	72.3	-3.25
2002	87.9	-1.71	71.7	-0.79
2003	74.6	-15.13	60.1	-16.16
2004	69.9	-6.37	54.6	-9.20
2005	70.6	1.09	54.6	-0.10
2006	64.9	-8.09	57.7	5.73
2007	67.7	4.23	57.6	-0.14
2008	59.6	-11.89	49.8	-13.53
2009	54.6	-8.38	52.0	4.35
2010	50.3	-7.90	49.5	-4.96
2011	55.9	11.13	56.3	13.61
2012	56.9	1.84	55.7	-0.94
2013	43.5	-23.63	41.1	-26.30

Table S3: Harvest dates of the Gi-FACE experiment. In this table, the harvest dates (start date and end date) of both spring harvest (H1) and summer harvest (H2) are shown.

Year	H1<br>Harvest Start	H1<br>Harvest End	H2<br>Harvest Start	H2<br>Harvest End
1998	15th Jun	16th Jun	3rd Sep	7th Sep
1999	14th Jun	14th Jun	25th Aug	31st Aug
2000	23rd May	23rd May	11th Sep	11th Sep
2001	28th May	28th May	10th Sep	10th Sep
2002	3rd Jun	3rd Jun	9th Sep	9th Sep
2003	19th May	19th May	8th Sep	8th Sep
2004	1st Jun	1st Jun	6th Sep	6th Sep
2005	13th Jun	13th Jun	13th Sep	13th Sep
2006	29th May	29th May	11th Sep	11th Sep
2007	30th May	30th May	10th Sep	10th Sep
2008	27th May	27th May	8th Sep	8th Sep
2009	25th May	25th May	7th Sep	7th Sep
2010	25th May	25th May	6th Sep	6th Sep
2011	23rd May	23rd May	5th Sep	5th Sep
2012	29th May	29th May	3rd Sep	3rd Sep
2013	3rd Jun	3rd Jun	2nd Sep	2nd Sep

Table S4: Data derived from the growing period of each year, for spring. Detailed data for better characterization of the properties of growing period are shown. For spring, six columns of data including the Day Of Year (DOY) of the last hard frost event, the DOY of the start of growing season, the number of days with Tmean<0ºC, the number of rain free days, the number of rain free consecutive days, as well as the true CO_2 Enrichment level, are presented in the table. "-" means there was no hard frost event in the corresponding year.

Year	Hard Frost (DOY)	Start of Growing Season	Num of Days Tmean<0ºC	Num of Rain free Days	Rain free Consecutive Days	CO_2 Enrichment (%)
1998	35	43	0	67	21	no data
1999	-	50	1	60	7	17.73
2000	28	29	1	48	8	19.12
2001	56	36	3	51	9	21.05
2002	6	20	0	64	21	21.92
2003	12	20	25	70	19	19.23
2004	-	32	12	80	14	17.77
2005	60	42	19	66	7	23.70
2006	24	47	14	47	9	21.45
2007	26	29	0	70	33	24.42
2008	-	53	2	37	11	25.22
2009	16	57	0	47	18	26.45
2010	67	54	7	47	11	27.85
2011	54	35	6	77	14	21.06
2012	43	48	0	63	15	8.46
2013	74	29	26	60	18	10.61

Table S5: Data derived from the growing period of each year, for summer. Detailed data for better characterization of the properties of growing period are shown. For summer, five columns of data including the DOY of the start of growing season, the number of days with Tmax>30ºC, the number of rain free days, the number of rain free consecutive days, as well as the true CO_2 enrichment level, are presented in the table.

Year	Start of Growing Season	Num of Days Tmax>30ºC	Num of Rain free Days	Rain free Consecutive Days	CO_2 Enrichment (%)
1998	166	6	45	17	no data
1999	165	2	45	12	17.55
2000	143	4	46	10	17.92
2001	148	7	56	14	18.97
2002	154	4	54	9	19.60
2003	139	17	64	11	16.44
2004	152	4	38	13	19.22
2005	164	3	46	12	21.91
2006	149	13	59	11	21.70
2007	150	3	50	14	19.76
2008	149	3	50	7	21.82
2009	148	3	52	8	23.44
2010	143	9	51	14	28.71
2011	144	3	43	7	24.93
2012	151	6	40	7	7.62
2013	155	9	61	18	48.53
